# Energy Types of Snoring Sounds in Patients with Obstructive Sleep Apnea Syndrome: A Preliminary Observation

**DOI:** 10.1371/journal.pone.0053481

**Published:** 2012-12-31

**Authors:** Li-Ang Lee, Jen-Fang Yu, Yu-Lun Lo, Yen-Sheng Chen, Ding-Li Wang, Chih-Ming Cho, Yung-Lun Ni, Ning-Hung Chen, Tuan-Jen Fang, Chung-Guei Huang, Hsueh-Yu Li

**Affiliations:** 1 Department of Otolaryngology, Sleep Center, Chang Gung Memorial Hospital, Chang Gung University, Taipei, Taiwan; 2 Graduate Institute of Medical Mechatronics, Taiouan Interdisciplinary Otolaryngology Laboratory, Chang Gung University, Taoyuan, Taiwan; 3 Department of Thoracic Medicine, Sleep Center, Chang Gung Memorial Hospital, Chang Gung University, Taipei, Taiwan; 4 Department of Pathology, Chang Gung Memorial Hospital, Chang Gung University, Taipei, Taiwan; University of Adelaide, Australia

## Abstract

**Background:**

Annoying snore is the principle symptom and problem in obstructive sleep apnea syndrome (OSAS). However, investigation has been hampered by the complex snoring sound analyses.

**Objective:**

This study was aimed to investigate the energy types of the full-night snoring sounds in patients with OSAS.

**Patients and Method:**

Twenty male OSAS patients underwent snoring sound recording throughout 6 hours of in-lab overnight polysomnogragphy. Snoring sounds were processed and analyzed by a new sound analytic program, named as Snore Map®. We transformed the 6-hour snoring sound power spectra into the energy spectrum and classified it as snore map type 1 (monosyllabic low-frequency snore), type 2 (duplex low-&mid-frequency snore), type 3 (duplex low- & high-frequency snore), and type 4 (triplex low-, mid-, & high-frequency snore). The interrator and test-retest reliabilities of snore map typing were assessed. The snore map types and their associations among demographic data, subjective snoring questionnaires, and polysomnographic parameters were explored.

**Results:**

The interrator reliability of snore map typing were almost perfect (*κ* = 0.87) and the test-retest reliability was high (*r* = 0.71). The snore map type was proportional to the body mass index (*r* = 0.63, *P* = 0.003) and neck circumference (*r* = 0.52, *P* = 0.018). Snore map types were unrelated to subjective snoring questionnaire scores (All *P*>0.05). After adjustment for body mass index and neck circumference, snore map type 3–4 was significantly associated with severity of OSAS (*r* = 0.52, *P* = 0.026).

**Conclusions:**

Snore map typing of a full-night energy spectrum is feasible and reliable. The presence of a higher snore map type is a warning sign of severe OSAS and indicated priority OSAS management. Future studies are warranted to evaluate whether snore map type can be used to discriminate OSAS from primary snoring and whether it is affected by OSAS management.

## Introduction

Snoring is the most prevalent symptom and also a principal indicator of obstructive sleep apnea syndrome (OSAS): 51.9% of male Taiwanese individuals older than 15 years having habitual snoring [1], and OSAS patients suffering loud snore [2], [3]. Accordingly, there were numberous studies aimed to rate the severity of snoring. Subjective questionaires, such as visual analogue scale (VAS), snoring severity score [4], snoring outcome survery (SOS) & spouse/bed partners survey (SBPS) [5], and snoring symptoms inventory [6], are freqently applied. Objective snoring index (SI), snoring sound intensity [2], and power spectrum [7] are often measured. Unfortunately, perception of snoring is highly subjective and usually lacked a strong association between its subjective and objective measurements [8]–[10]. Due to limited information of solitory SI provieded by polysomnography (PSG), the researchers have developed some more sophisticated methods to measure snoring and correlate it with the underlying physiological abnormalities responsible for snoring [3], [7], [11].

**Figure 1:**
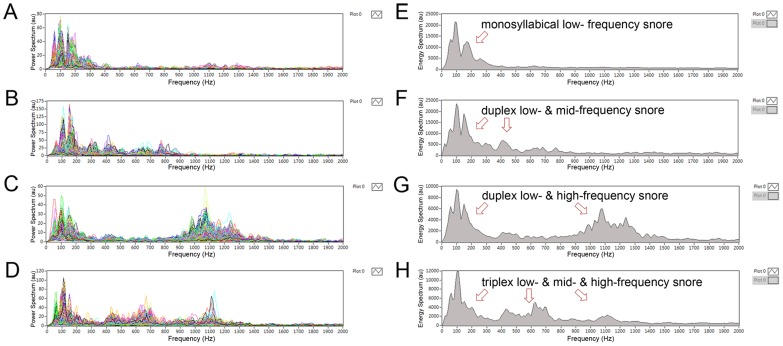
Spectral analyses of full-night snoring sounds including power spectra of overall snore events (A–D) and energy spectrum of an overnight snore map (E–H). (A) & (E) Primarily monosyllabical low-frequency snoring (type 1 snore map). (B) & (F) Duplex low- and mid-frequency snores (type 2). (C) & (G) Duplex low- and high-frequency snoring (type 3). (D) & (H) Triplex low-, mid-, and high-frequency snores (type 4).

The power spectrum is a method to simultaneously measure the snore inttensity and frequency. Herzog *et al.* demonstrated that simple snorer have peak intensities between 100 and 300 Hz and OSAS patients have peak intesities above 1,000 Hz [7]. Fiz *et al.* analyzed snoring during full-night PSG and indicated that the spectral energy of snores become constrated at lower frequencies (100 Hz–500 Hz) in OSAS patients with AHI ≥15 [3]. Since 2011, we had developed a specially developed software program, named as Snore Map® currently, to analyze the full-night snoring sounds [12]. In our previous study, we found that there were three characteristic snoring sounds existing in the power spectrum and their sound duration was related to the specific vibrator: the soft palate has a longest snoring duration, followed by the epiglottis and tongue base. However, most of the OSAS patients have concurrent three snoring sounds in their natural sleep but the compositions of snoring sounds are so complicated to be interpreted (Fig. 1A–1D).

According to the concept of “Energy = power × time”, we counted the 6-hour powers of different frequency snoring sounds and found that the patients’ snoring energy types were more easily to be classified. Herein, we defined four energy types (snore map types) of snoring sounds in OSAS patients: type 1 (monosyllabical low- frequency snore, Fig. 1E), type 2 (duplex low- & mid-frequency snore, Fig. 1F), type 3 (duplex low- & high-frequency snore, Fig. 1G), and type 4 (triplex low- & mid- & high-frequency snore, Fig. 1H). In the present study, we introduced our new snoring sound analytical method and performed reliablity tests. Moreover, we also explored the associations among snore map types, demographic characterics, subjective snoring questionnaire scores, and PSG data in male patients with OSAS.

## Materials and Methods

### Patients

Twenty consecutive male adult patients with a history of habitual snoring and witnessed sleep apnea, and/or excessive daytime sleepiness were prospectively enrolled from the Sleep Center of the Chang Gung Memorial Hospital in Taoyuan, Taiwan. They had undergone a snoring sound recording accompanied with a standard overnight PSG. Primary inclusion criterion was apnea-hypopnea index (AHI) >5, and secondary criterion was fitting in with snore map type: type 1 (*n* = 5), type 2 (*n* = 5), type 3 (*n* = 5), and type 4 (*n* = 5). Their primary exclusion criteria were history of anti-snoring treatment and habitual using sedative or hypnotic medications. The body mass index (BMI), neck circumference, tonsil size, and tongue position were measured according to Friedman’s classification [13]. The anatomic staging method had been described in detail elsewhere [14]. Table 1 demonstrates their demographic characteristics, snoring questionnaire scores, PSG parameters, and snore map types. The Institutional Review Board of the hospital approved the study and informed consent was obtained from each subjects.

**Table 1:** Demographic characteristics, snoring questionnaires, polysomnographic parameters, and snore map type.

	Demographic characteristics						Snoring questionnaires			Polysomnographic parameters				Severity of OSAS	Snore map type
No.	Age (yr)	BMI (kg/m^2^)	NC (cm)	Tonsil size	FTP	Stage	VAS	SOS	SBPS	AHI	AI	HI	SI		
1	44	24.3	37	2	II_b_	2	10	39	33	38.5	0.2	38.3	19.8	2	2
2	46	25.5	40	2	II_b_	2	8	49	39	22.7	11.8	10.9	4.4	1	1
3	39	26.2	41	1	I	2	5	53	50	57.0	9.1	47.9	0.7	2	4
4	27	25.3	38	2	II_a_	2	8	41	33	10.3	0	10.3	2.0	1	1
5	47	31.6	42	2	II_a_	2	8	44	33	26.3	0	26.3	180.5	1	2
6	49	28.2	42	2	II_a_	2	9	41	33	57.5	5.8	51.7	188.6	2	3
7	26	28.1	38	2	II_a_	2	10	18	17	32.3	6.5	25.8	110.0	2	3
8	39	27.5	39	2	II_a_	2	6	56	50	62.6	26.0	36.6	263.3	3	3
9	46	29.8	41	3	II_a_	2	8	21	22	51.4	43.8	7.6	309.6	2	4
10	54	27.7	42	3	III	3	9	28	28	54.8	54.1	0.7	214.8	2	4
11	20	28.7	40	3	I	1	8	28	28	45.0	0.4	44.6	332.7	2	4
12	38	31.2	39	2	II_a_	2	8	30	28	104.0	13.9	90.1	520.1	3	4
13	41	23.8	38	1	II_b_	2	10	23	17	53.1	34.7	18.4	320.3	2	1
14	53	24.3	37	1	II_b_	2	8	64	17	41.3	7.2	34.1	128.5	2	1
15	47	25.7	38	2	III	3	10	31	17	74.8	5.3	69.5	51.0	3	1
16	46	24.2	37	3	II_b_	2	6	30	50	59.8	54.3	5.5	251.4	2	2
17	28	31.1	43	3	III	2	8	46	33	77.3	70.9	6.4	148.9	3	3
18	39	23.3	36	3	III	2	7	41	39	25.8	0.2	25.6	11.3	1	2
19	39	29.3	44	3	II_b_	2	5	39	44	73.5	31.4	42.1	94.5	3	3
20	28	27.8	40	2	II_b_	2	10	25	22	16.3	0	16.3	493.7	1	2

BMI: body mass index. FTP: Friedman tongue position. NC: neck circumference. VAS: visual analogue scale. SOS: snoring outcome survey. SBPS: spouse/bed partners survey. AHI: apnea-hypopnea index. AI: apnea index. HI: hypopnea index. SI: snoring index. OSAS: obstructive sleep apnea syndrome.

### Snoring Survey

All patients completed three snore outcome measures: the VAS, SOS, and SBPS questionnaires. The patients were asked to quantify the average intensity of their snoring using a VAS from 0 (no snoring) to 10 (very severe snoring). The SOS and SBPS are two valid, reliable, and disease-specific outcome measures. The SOS comprised of eight Likert-type items to comprehensively evaluate the duration, loudness, and frequency of snoring, and the SBPS containing another three Likert-type items. The SOS and SBPS were normalized on a scale ranging from 0 (worst) to 100 (best) [5]. A Mandarin Chinese version of the SOS and SBPS was used with permission in this study [9].

### Sleep Study

Standard overnight PSGs (Nicolet UltraSom System, Madison, WI, USA) were performed in the sleep laboratory to document sleep parameters in each patient. The PSG parameters used in this study were snoring index (PSG-SI), AHI, apnea index (AI), and hypnonea index (HI). All respiratory events were defined as previously described [15]. Apnea was defined as a drop in the peak thermal sensor excursion by at least 90% of baseline for at least 10 seconds. Hypopnea was defined as a decrease ≥30% in the nasal pressure signal excursions for at least 10 seconds accompanied by desaturation of 4% or more from pre-event baseline or an arousal from sleep. The PSG studies were manually scored by an experienced pulmonologist who was blinded to the status of the patients. Patients were further categorized as having mild-moderate (AHI <30), severe (AHI, 30–59), or very severe (AHI ≥60) OSAS and placed in respective groups in the present study because there were 30% of the OSAS patients with AHI ≥60 at our sleep center.

### Snore Map

We used three external measurement microphones (TEDS type 46AE, G.R.A.S. Corp., Holte, Denmark), positioned 100 cm above the patient’s head [12], [16], to record snoring sounds at the sleep laboratory. We only adopted the snoring sound signal acquired by the central microphone. The other two microphones, placed bilaterally, were the reference for monitoring the snoring signal acquired once the subject faced to right-hand side, or faced to left-hand side for confirming the signal quality by the central microphone was sufficient for analysis after comparison among these three microphones.

The microphone detected sound frequencies between 3.15 and 20,000 Hz. A sound pressure level calibration was performed using a sound calibrator (B&K4321, Brüel & Kjær Corp., Nærum, Denmark) before each test. The intensities of the recorded snoring sounds were collected by portable data cards (PXI 4462, National Instruments Corp., Austin, TX, USA) and processed by digital recording software (Sound & Vibration Toolkit for Labview, National Instruments Corp., Austin, TX, USA) at sample rate of 44,100 Hz. The frequency power spectrum was created by fast Fourier transformation (range, 3.15 Hz–2,000 Hz).

A snore detection system was designed to separate the snore episodes from environmental (machines, door opening, moving furniture, etc.) and other biological noises (body movements, oral communications, coughs, etc.).

First, recording the environmental sounds of 10 minutes in a study room (silent sleep laboratory) were sampled and analyzed at the beginning of each test, and we found the highest intensity of background noises were relatively constant and occurred between 3.15 Hz and 40 Hz. Using a high-pass filter technique, the interference of background noises and snore episodes was reduced.

**Figure 2:**
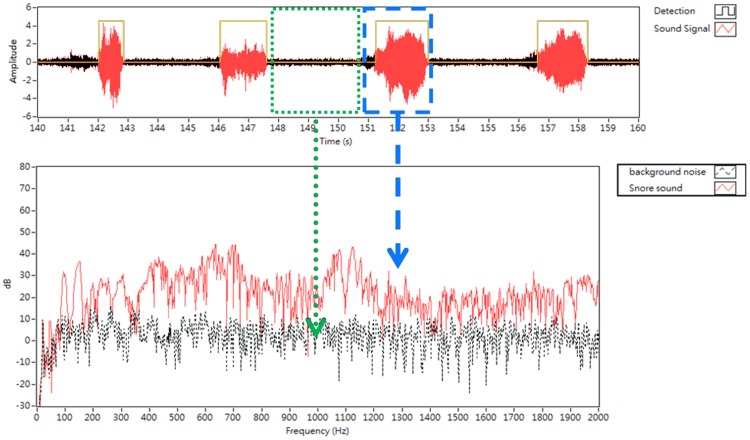
The spectrum of snoring sound (red line) and background noise (black line). Note that the duration of snoring sound was between 0.6 and 4.0 seconds (blue box) and noise intensity in sound pressure level was general less than 10 dB when frequencies were higher than 450 Hz (green box).

Second, a snoring sound detection algorithm was designed based on an adaptive energy threshold. In our previous study [12], we found the sound durations of audible snores were different from other biological noises (snores, 0.6 second–3.7 seconds; body movements, oral communications, ≥4.0 seconds; cough, <0.5 second). The average duration of snoring generated at the soft palate, epiglottis, and tongue base were 3.7 seconds, 2.2 seconds, and 1.29 seconds, respectively. Moreover, energies of snores (>0.05 au) were usually higher than energies of environmental noises (<0.02 au). For an all-night analysis of snoring signals, an automatic detection algorithm is implanted base on these two criteria: (1) sound energy higher than 0.05 au and (2) sound duration between 0.6 second and 4.0 second. These “meaningful snoring events” were further analyzed in the present study (Fig. 2).

Third, the sensitivity, positive predictive value, and stability of this system for the detection of snores were evaluated. We compared the detection results of ten different 90-minute snoring sounds tested by this system (total detected snore number, 3857) with those snore episodes manually scored by agreement among five careful listeners (total snore number, 3826). The sensitivity and positive predictive value of our detection method were 99.9% and 99.1%, respectively. Moreover, this system was evaluated by the Jury stability test and was considered as “stable” for detecting snores [17].

**Figure 3:**
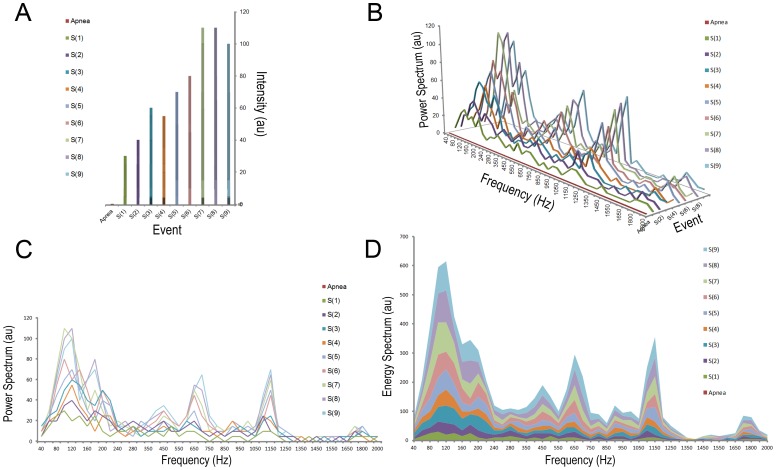
An example of an energy spectrum construction. (A) Sound intensities of the nine detected snores after an apnea event within one minute. (B) Three dimensional figure showing the power spectrum of each snoring episode. (C) Two dimensional figure showing nine power spectra together. (D) Energy spectrum strengthening the contributions of the predominately loud snoring sounds and helping us to type the snore map.

We simultaneously recorded snoring sounds and PSG for 6 hours during each subject’s natural sleep. We harmonized the time scale and correlated respiratory events and snoring episodes manually by adjusting the time axis of both systems. Figure 3A demonstrates an apnea episode followed by nine snoring events within one minute. A snoring power spectrum demonstrated a snapshot of one snoring event and we further collected a series of snoring power spectral analyses at different times and displayed them using a three dimensional snoring power spectrum (Fig. 3B) and subsequently transformed it into a two dimensional snoring power spectrum (Fig. 3C). The overall energy of each frequency unite was further calculated and plotted in the frequency energy spectrum (Fig. 3D).

**Figure 4:**
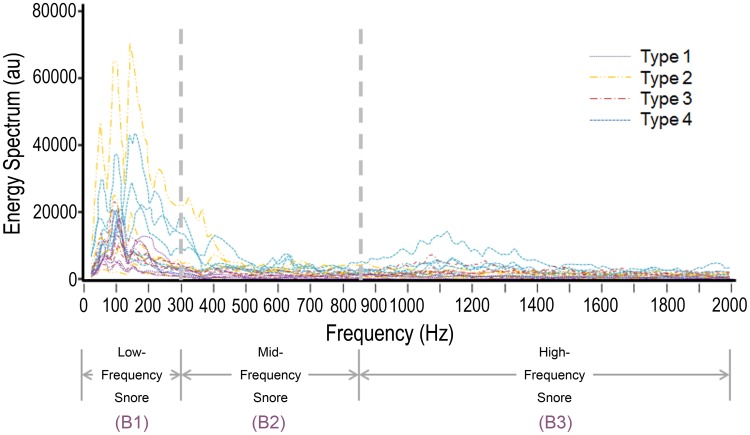
Energy spectrum of the 20 subjects categorized by energy typing showing their exact distributions of snoring sound energy. Some cases with low amplitude of snoring sound energy needed adjustment of the Y-axis scales for an easier typing.

The previously most popular definition of high-frequency domain of the post-apneic snore was above 800 Hz [18]. However, that definition was based on two microphones, one attached to the sternum and one placed 15 cm above the bead. In this study, we tried to categorize all the noise signals of the full-night snores with and various frequency domains. Accordingly, we defined our reference band widths of low-frequency, middle-frequency, and high-frequency snoring by direct observations of the power spectrum of 20 OSAS subjects. The lead author, aware of subject identity throughout, performed all snore map examinations, and the power spectrums were later reviewed concurrently but independently by two authors. The blinded author had no knowledge of history or physical examination findings, or sleep study results. We decided the upper limit (Line 1, 292±50 Hz; coefficient of variance [CV], 17.1%) to include most of the low-frequency snores, and the lower limit (Line 2, 866±70 Hz; CV, 8.1%) to include the high-frequency snores if presenting or the mid-frequency snores in subjects without high-frequency snores for each patient separately. For convenience, we defined the Line 1 as 300 Hz and the Line 2 as 850 Hz. Accordingly, there were three different bands (B) as follows: B1 (low-frequency, 40 Hz–300 Hz), B2 (mid-frequency, 301 Hz–850 Hz), and B3 (high-frequency, 851 Hz–2,000 Hz) (Fig. 4).

For snore map typing, we adjusted the highest energy to the upper limit of the Y-axis (Fig. 1E–H) due to a better observation of the other bands. We performed an interrater reliability test of snore map typing by two authors: one unblinded author and one blinded author. Moreover, we also underwent a test-retest reliability test of Line 1 determining, Line 2 determining, and snore map typing two months later by the same blinded author.

We further analyzed each snore and obtained three independent variables in a 6-hr snoring record: total snoring index (total-SI), total maximal sound intensity (total-Imax [dB]), and total mean sound intensity (total-Imean [dB]). Using frequency filter programs, we calculated the B1-SI, B1-Imax, B1-Imean, B1 peak sound frequency (B1-Fpeak [Hz]) and B1 mean sound frequency (B1-Fmean [Hz]), the B3-SI, B3-Imax, B3-Imean, B3-Fpeak, and B3-Fmean, and the B2-SI, B2-Imax, B2-Imean, B2-Fpeak, and B2-Fmean. Data for each of these acoustic parameters were averaged for all the detected episodes.

### Statistical Analysis

Descriptive statistics were calculated for baseline subject characteristics and results are reported with mean ± standard deviation. Summary statistics for the cut-off frequencies (Lines 1 and 2) of the snore spectrum and the snore map types were also calculated with the *Kappa* statistic for paired proportions to determine consistency among raters. A Mann-Whitney *U* test for independent groups was used for between-group comparisons of sound parameters. A Kruskal-Wallis test was used for among-group comparisons of sound parameters. The PSG-SI and SI calculated by the Snore Map® were compared by using a paired Student *t*-test. A Spearman nonparametric correlation test and/or an adjusted partial correlation test were used to investigate the test-retest reliability, and the relationships of snore map types, clinical factors, and acoustic parameters. Two-sided *P* values <0.05 were considered statistically significant.

## Results

### Characterization of Patients

Table 2 summarizes the demographic characters, snoring questionnaires, PSG parameters in the different snore map type groups. We investigated if any significant difference among the four snore map types. The BMI was lowest in the snore map type 1 (*P* = 0.026), whereas the other demographic characteristics, snoring questionnaire scores, PSG parameters, and severity of OSAS were similar in our subjects (All *P*>0.05). Patients with snore map type 1 seemed to have a lower neck circumference than the patients with type 3 or type 4; however, the difference did not reach a statistical significance among these four groups.

**Table 2:** Demographic characteristics, snoring questionnaires, and polysomnographic parameters among four snore map types.

	All cases	Type 1	Type 2	Type 3	Type 4	*P* value†	*P* value between types‡					
	(*n* = 20)	(*n* = 5)	(*n* = 5)	(*n* = 5)	(*n* = 5)	Total	1–2	1–3	1–4	2–3	2–4	3–4
Demographic characteristics												
Age	38±9.1	42.8±9.8	40.8±7.8	36.2±9.4	39.4±12.6	0.681	0.599	0.249	0.530	0.396	0.753	0.751
BMI (kg/m^2^)	26.9±2.8	24.9±0.8	26.2±3.5	28.8±1.4	28.7±1.9	0.040*	1.00	0.009*	0.009*	0.175	0.251	0.917
NC (cm)	39.5±2.2	38.1±1.1	38.4±2.5	41.1±2.5	40.5±0.8	0.071	0.670	0.045*	0.020*	0.093	0.206	0.528
Tonsil Size	2.2±0.7	1.6±0.5	2.4±0.5	2.4±0.5	2.4±0.9	0.182	0.058	0.058	0.121	01.00	0.817	0.817
FTP	2.7±0.9	3.0±0.7	3.0±0.7	2.6±0.9	2.0±1.2	0.278	1.00	0.371	0.131	0.371	0.131	0.262
Stage	2.1±0.4	2.2±0.4	2.0±0.0	2.0±0.0	2.0±0.7	0.808	0.317	0.317	0.606	1.00	1.00	1.00
Snoring questionnaires												
VAS	8.1±1.6	8.8±1.1	8.2±1.8	7.6±2.1	7.6±1.5	0.684	0.504	0.382	0.238	0.595	0.665	0.828
SOS	37.3±12.5	41.6±16.1	35.7±8.3	40.2±13.8	31.8±12.3	0.599	0.602	0.917	0.251	0.402	0.465	0.347
SBPS	31.7±11.3	24.5±10.8	35.6±10.1	35.6±12.8	31.1±10.8	0.352	0.133	0.190	0.335	1.00	0.337	0.395
Polysomnographic parameters												
AHI	49.2±23.4	40.4±25.3	33.3±16.8	60.6±17.7	62.4±23.7	0.119	0.754	0.175	0.175	0.047*	0.076	0.465
AI	18.8±22.0	11.8±13.5	10.9±24.2	28.1±26.5	24.3±23.3	0.210	0.340	0.347	0.251	0.074	0.115	0.917
HI	30.4±23.0	28.6±24.8	22.4±12.3	32.5±17.3	38.2±36.0	0.810	0.917	0.602	0.917	0.251	0.465	0.754
SI	182.3±157.0	101.2±132.7	191.3±198.1	161.0±67.8	275.6±189.4	0.432	0.347	0.251	0.251	0.917	0.465	0.175
OSAS severity	2.0±0.7	1.8±0.8	1.4±0.5	2.6±0.5	2.2±0.4	0.055	0.419	0.151	0.421	0.032	0.095	0.310

BMI: body mass index. FTP: Friedman tongue position. NC: neck circumference. VAS: visual analogue scale. SOS: snoring outcome survey. SBPS: spouse/bed partners survey. AHI: apnea-hypopnea index. AI: apnea index. HI: hypopnea index. SI: snoring index. OSAS: obstructive sleep apnea syndrome.

† Significance was assessed by a Kruskal Wallis test.

‡ Significance was tested using a 2-sided Mann-Whitney *U* test.

* A value of *P*<0.05 was considered significant.

### Interrater and Test-retest Reliabilities of Snore Map Typing

In our new snoring sound analytic method, the interrater reliabilities for the raters were found to be significant to determine the Line 1 (*Kappa* = 0.66, *P*<0.001), the Line 2 (*Kappa* = 0.49, *P*<0.001), and the snore map typing (*Kappa* = 0.87, *P*<0.001). Interestingly, the typing of the snore map had a greater reliability than for the determinations of the cut-off values of Lines 1 and 2. The test-retest reliabilities for the same rater were significant to determine the Line 1 (*r* = 0.60, *P* = 0.005), the Line 2 (*r* = 0.85, *P*<0.001), and the snore map typing (*r* = 0.71, *P* = 0.001). Accordingly, we could classify the snore map type reliably.

### Acoustic Comparison among Four Snore Map Types

Figure 1 demonstrates that the proportions of low-frequency, mid-frequency, and high-frequency snoring sound energies were apparently different among four snore map types. We further performed the power spectral analyses of the snoring and demonstrated the key parameters in Table 3. Firstly, the B1-SI were indifferent to the PSG-SI whereas the total-SI, B2-SI, and B3-SI were significantly higher than the PSG-SI (*P* = 0.014, 0.001 & <0.001 respectively) in all cases. In general, patients with a higher grade of snore map type had higher total-Imax, total-Imean, and B3-Imean (All *P*<0.05). Subjects with snore map type 1 had the lowest total-Imax, total-Imean, B2-Imax, B2-Imean, and B3-Imean among these four groups, whereas type 4 patients had the highest total-Imax, total-Imean, B2-Imax, B2-Imeaan, and B3-Imean (All *P*<0.05). Snore map type 3 cases had the lowest B3-Fpeak compared with other three type patients (*P* = 0.037).

**Table 3:** Snoring power spectral data among four snore map types.

		Type 1	Type 2	Type 3	Type 4	*P* value†	*P* value between types‡					
		(*n* = 5)	(*n* = 5)	(*n* = 5)	(*n* = 5)	Total	1–2	1–3	1–4	2–3	2–4	3–4
Total	SI	273.8±217.3	401.6±336.5	200.6±78.8	373.2±134.8	0.346	0.602	0.917	0.251	0.249	0.917	0.047*
	Imax (dB)	64.9±6.1	70.7±6.4	72.3±4.5	78.9±5.3	0.014*	0.175	0.076	0.009*	0.465	0.047*	0.028*
	Imean (dB)	50.2±4.4	52.6±3.2	56.5±3.9	59.5±3.8	0.018*	0.465	0.047*	0.016*	0.076	0.028*	0.251
	Fpeak (Hz)	120.0±27.4	128.0±29.5	116.0±194.5	122.0±43.8	0.657	0.277	0.658	1.000	0.501	0.381	0.435
	Fmean (Hz)	726.0±554.0	832.0±530.2	1370.0±242.4	1668.0±436.8	0.072	0.465	0.142	0.076	0.094	0.028*	0.465
B1	SI	261.6±222.9	375.0±347.0	163.0±69.8	304.4±146.1	0.595	0.754	0.465	0.602	0.465	0.917	0.117
	Imax (dB)	59.0±3.5	63.1±5.1	60.0±4.6	66.7±6.6	0.173	0.117	0.754	0.094	0.175	0.347	0.175
	Imean (dB)	46.1±2.1	47.9±4.2	47.4±1.0	48.8±3.7	0.488	0.465	0.251	0.251	0.917	0.602	0.175
	Fpeak (Hz)	250.0±34.6	270.0±22.3	236.0±53.7	252.0±40.9	0.661	0.290	0.672	0.915	0.287	0.462	0.673
	Fmean (Hz)	112.7±18.2	137.3±22.3	116.1±17.4	116.5±37.9	0.216	0.076	0.754	0.917	0.117	0.117	0.465
B2	SI	255.2±139.0	418.8±271.3	231.4±82.0	418.2±114.7	0.127	0.251	0.917	0.117	0.117	0.917	0.028*
	Imax (dB)	58.0±7.3	68.2±5.4	61.9±2.6	76.4±6.5	0.007*	0.047*	0.1117	0.016*	0.076	0.076	0.009*
	Imean (dB)	44.3±3.2	47.9±3.9	45.2±1.2	51.6±4.6	0.044*	0.251	0.251	0.016*	0.347	0.175	0.028*
	Fpeak (Hz)	828.0±49.2	786.0±73.3	844.0±8.9	848.0±4.5	0.126	0.118	0.700	0.881	0.105	0.044*	0.439
	Fmean (Hz)	424.9±56.2	430.6±97.7	413.2±71.2	468.9±89.7	0.832	0.917	0.917	0.602	0.754	0.602	0.251
B3	SI	358.0±133.0	327.8±204.4	363.2±48.5	555.6±138.6	0.069	0.917	0.754	0.047*	0.917	0.076	0.009*
	Imax (dB)	61.9±10.8	66.1±8.9	72.0±4.5	76.9±4.8	0.064	0.465	0.175	0.028*	0.347	0.076	0.076
	Imean (dB)	43.3±4.1	44.2±4.1	49.4±4.7	52.8±3.3	0.011*	0.602	0.076	0.009*	0.117	0.009*	0.175
	Fpeak (Hz)	1992.0±13.0	1950.0±111.8	1734.0±267.1	1980.0±28.3	0.037*	0.700	0.020*	0.638	0.041*	0.700	0.026*
	Fmean (Hz)	1209.9±158.9	1242.9±178.2	1215.9±124.2	1237.8±81.6	0.881	0.754	0.465	0.754	0.465	0.602	0.465

SI: snoring index. Imax: maximal sound intensity. Imean: mean sound intensity. Fpeak: peak frequency. Fmean: mean frequency. B1: the band between 40 Hz and 300 Hz. B2: the band between 301 Hz and 850 Hz. B3: the band between 851 Hz and 2000 Hz.

† Significance was assessed by a Kruskal Wallis test.

‡ Significance was tested using a 2-sided Mann-Whitney *U* test.

* A value of *P*<0.05 was considered significant.

The differences in the B2-Imax between type 1 and type 2 reached a statistical significance. Type 2 had a significant higher B3-Fpeak than type 3. Type 3 and type 4 were significantly different in the total-SI, B1-SI, B2-SI, B3-SI, and B2-Imax. Type 3 patients had higher mean total-Imean B2-Fpeak, and lower B3-Fpeak than type 1 patients. The total-Imax, total-Imean, B2-Imax, B2-Imean, B3-SI, B3-Imax, and B3-Imean were particularly higher of type 4 patients than type 1 patients. Type 2 and type 4 were different in the total-Imax, total-Imean, total-Fmean, B2-Fpeak, and B3-Imean.

### Associations of Snore Map Type, Demographic Characteristics, Snoring Questionnaires, and PSG Parameters

**Figure 5:**
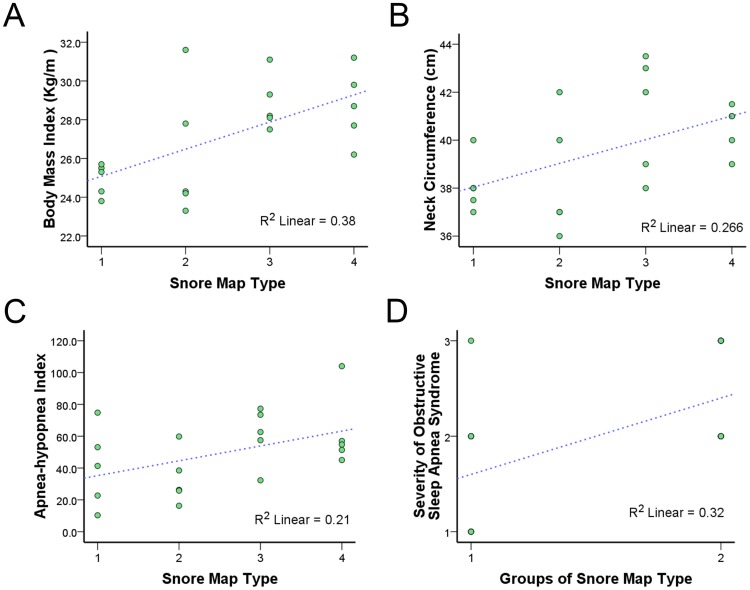
Scatter plots of clinical parameters versus snore map type. (A) A scatter plot of body mass index (BMI) versus snore map type. (B) A scatter plot of neck circumference versus snore map type. (C) A scatter plot of apnea-hypopnea index versus snore map type. (D) A scatter plot of severity of obstructive sleep apnea syndrome (OSAS) versus groups of snore map type. After adjustment for BMI and neck circumference, snore map type 3–4 was significantly associated with severity of OSAS (*R* ^2^ = 0.28, *P* = 0.026). Blue dashed line indicates linear fit line.

Figure 5A demonstrates scatter plots of BMI versus snore map type and indicates that sore map types were significantly correlated to BMI (*r* = 0.63, *P* = 0.003). Figure 5B shows scatter plots of neck circumference versus snore map type and reveals a significant correlation between sore map types and neck circumference (*r* = 0.52, *P* = 0.018). Scatter plots of AHI versus snore map type indicates that snore map types were significantly correlated to AHI (*r* = 0.46, *P* = 0.040, Fig. 5C). The snore map type was unrelated to the age, tonsil size, tongue position, Friedman’s stage, VAS, SOS, and SBPS.

We further explored the associations of acoustic parameters and subjective questionnaire scores among these OSAS patients. After adjustment for BMI and neck circumference, the total-Fpeak was independently associated with the VAS score (*r* = 0.48, *P* = 0.046) and the total-Imean was independently associated with the SOS score (*r* = –0.57, *P* = 0.013). The B2-Fpeak was associated with the SBPS (*r* = –0.49, *P* = 0.029), however, their association become insignificant after adjustment for BMI and neck circumference. Moreover, the VAS, SOS, and SBPS scores were not associated to the AHI.

We further categorized the subjects into the snore map type 1–2 group and the snore map type 3–4 group (Table 4). The snore map type 3–4 group had significantly higher BMI, neck circumference, AHI, AI, and severity of OSAS than the snore map type 1–2 group. Among acoustic parameters, the snore map type 3–4 group had remarkably higher total-Imax, total-Imean, B3-Imax, and B3-Imean. After adjustment for BMI and neck circumference, snore map type 3–4 was marginally significantlycorrelated with AHI (*r* = 0.44, *P* = 0.065), but was significantly associated with severity of OSAS (*r* = 0.52, *P* = 0.026, Fig. 5D). Moreover, we adjusted for BMI and neck circumference, snore map type 3–4 was significantly related to total-SI (*r* = –0.631, *P* = 0.005), total-Imean (*r* = 0.49, *P* = 0.041), total-Fmean (*r* = 0.59, *P* = 0.009), B1–SI (*r* = –0.64, *P* = 0.004), and B2–SI (*r* = –0.59, *P* = 0.011). Interesting, the AHI was significantly associated snore map type 3–4 (*r* = 0.54, *P* = 0.014), total-Imax (*r* = 0.57, *P* = 0.009), total-Imean (*r* = 0.54, *P* = 0.014), total-Fmean (*r* = 0.79, *P*<0.001) and B3-Imean (*r* = 0.54, *P* = 0.014), whereas the AHI was still correlated significantly with the total-Imax (*r* = 0.49, *P* = 0.039), total-Fmean (*r* = 0.78, *P*<0.001), B3-Imax (*r* = 0.48, *P* = 0.042), and B3-Fmean (*r* = 0.53, *P* = 0.023) after adjustment for BMI and neck circumference.

**Table 4:** Comparison of demographic characteristics, snoring questionnaires, polysomnographic parameters, and acoustic parameters between the snore map type 1–2 group and the snore map 3–4 group.

Group	Demographic characteristics									Snoring questionnaires						Polysomnographic parameters								Severity of OSAS
	Age (yr)	BMI (kg/m^2^)		NC (cm)		Tonsil Size		FTP	Stage	VAS		SOS		SBPS		AHI		AI		HI		SI		
Type 1-2	41.8±8.4	25.6±2.5		38.3±1.8		2.0±0.7		3.0±0.7	2.1±0.3	8.5±1.4		38.6±12.4		30.0±11.5		36.9±20.6		11.4±18.5		25.5±18.7		146.3±165.9		1.6±0.7
Type 3-4	37.8±10.6	28.8±1.6		40.8±1.8		2.4±0.7		2.3±1.1	2.0±0.5	7.6±1.7		36.0±13.1		33.3±11.4		61.5±19.7		26.2±23.6		35.4±26.8		218.3±147.1		2.4±0.5
*P* †	0.287	0.005*		0.009*		0.185		0.081	0.584	0.287		0.677		0.645		0.019*		0.049*		0.364		0.257		0.014*
	**Acoustic parameters**																							
	**Total-SI**		**Total-Imax**		**Total-Imean**		**B1-SI**		**B1-Imax**		**B2-Imean**		**B2-SI**		**B2-Imax**		**B2-Imean**		**B3-SI**		**B3-Imax**		**B3-Imean**	
Type 1-2	337.7±275.4		67.8±6.7		51.4±3.8		318.3±281.3		61.0±4.7		47.0±3.2		337.0±220.8		63.1±8.1		46.1±3.9		342.9±163.3		64.1±9.6		43.8±3.9	
Type 3-4	286.9±138.2		75.6±5.8		58.0±3.9		233.7±131.2		63.4±6.4		48.1±2.7		324.8±136.1		69.1±8.9		48.4±4.6		459.4±141.0		74.5±5.1		51.1±4.2	
*P* †	0.940		0.010*		0.002*		0.705		0.571		0.326		1.000		0.151		0.151		0.131		0.023*		0.002*	

BMI: body mass index. FTP: Friedman tongue position. NC: neck circumference. VAS: visual analogue scale. SOS: snoring outcome survey. SBPS: spouse/bed partners survey. AHI: apnea-hypopnea index. AI: apnea index. HI: hypopnea index. SI: snoring index. OSAS: obstructive sleep apnea syndrome. Imax: maximal sound intensity. Imean: mean sound intensity. B1: the band between 40 Hz and 300 Hz. B2: the band between 301 Hz and 850 Hz. B3: the band between 851 Hz and 2000 Hz.

† Significance was assessed by a Kruskal Wallis test.

* A value of *P*<0.05 was considered significant.

## Discussion

This is the first study investigating OSAS patients’ snoring using the snoring energy spectrum. The findings suggest that there are at least four different energy types of the snoring sounds (snore map types) that are fairly associated with the severity of OSAS. The fine reliabilities of snore map measuring indicate that snore map typing represents a simple trustworthy way to classify the energy pattern of each OSAS patient’s snoring in nature sleep. Notably, snore map type cannot be differentiated by current subjective snoring questionnaires. These different distributions of snoring map may reflect the multiple vibrating sites of the upper airway, including contributions from the velum, tongue base, pharyngeal wall, and epiglottis.

Perez-Padilla and coworkers firstly described that most of the power of snoring noise was below 2,000 Hz, and patients with OSAS had residual energy at 1,000 Hz, whereas the nonapneic snorers did not [18]. They also recommended that the ratio of power above 800 Hz to power below 800 Hz of the first post-apneic snore could be used to separate simple snorers from patients with OSAS. In our study, we used a relatively long distance (100 cm) to measure the full-night snoring sounds. Not only the first post-apneic snore but also the remaining snores following apneic events were all analyzed. We found that snoring signs between 800 Hz and 850 Hz frequently continued to snoring signals below 800 Hz. Therefore, we categorized snores with the frequency domain from 850 Hz to 2000 Hz to “high-frequency snores”. The snore map type 3–4, containing full-night high-frequency snore energy, was associated with higher AHI and these results further supported Perez-Padilla’s findings.

Beck et al. found that there are two distinctly different patterns of snoring sound waveform by using power spectrum as follows: complex-waveform snore and simple-waveform snore. They found that brief airway closure induce collide of the airway walls and produce complex-waveform snores, whereas simple-waveform snores result from oscillation of a patent airway lumen [19]. Solà-Soler et al. used linear prediction autoregressive modeling with a low order for spectral envelope estimation of a piezoelectric contact sensor placed beside the cricothyorid notch [20]. They found the spectral envelope concentrated in a reduced number of formant distributions in simple snorers and indicated that the mechanism of snore production has some common characteristics. By contrast, the standard deviation of some snoring formant frequencies is significantly higher in OSAS patients than in simple snorers. As Fiz’s [3] and Ben-Israel’s [21] findings, the snore signals also became instable with various amplitudes and durations in apneic phase. However, most of the OSAS patients had various durations of snoring and different proportions of simple- and complex-waveform snores.

In our study, we tried to simplify the formant distribution, pitch density, and running variance of snoring sounds by snoring energy spectrum typing. For this purpose, we developed a semi-automatic graphical user interface to automatically detect full-night snoring energy and to manually decide which type of snore map is based on audio and visual perception. Snore map type 1 (Figure 1E) is composed of primary simple-waveform snore powers (Fig. 1A), and snore map type 4 (Figure 1H) consists of principle complex-waveform snoring powers of a 6-hour sleep (Figure 1D). This finding suggested that type 1 subjects seems to represent hypopneic predominate OSAS and type 4 patients may depict predominant apneic OSAS. Moreover, our observations suggested that a higher grade of snore map typing symbolizes a more complex sound energy form and a severer degree of OSAS. The presence of B3-energy is associated with a higher chance of transient airway closures with multiple generators of snoring and with the increasing AHI. Of note, our proposed method did not consider the time interval between snores that has been regarded as an important predicator of severe OSAS [22]. Nevertheless, the clinicians can easily and quickly decide which snoring patient is priority for standard PSG examination based on the snore map type.

Miyazaki et al. found that the lower frequency snoring sound (fundamental frequency [ff] = 102.8±34.9 Hz) is characteristic of the soft palate obstruction and the higher frequency snoring sound (ff = 331.7±144.8 Hz) resulting from the tonsil/tongue base obstruction according to the intraluminal pressure of the upper airway [23]. Agrawal et al. used sleep endoscopy to examine the site of vibration and sound frequency spectrum, and found that the tonsillar and palatal vibrations produce similar low-frequency snoring sound spectra (Fpeak = 170 Hz & 137 Hz, respectively) and tongue-base vibrations result in high-frequency snoring (Fpeak = 1,243 Hz) [24]. The mid-frequency snoring sound is the feature of epiglottic snores (ff = 249.4±79.7 Hz [18]; Fpeak = 490 Hz [22]). To our knowledge, OSAS patients often have different durations and multiple generators of snoring. Therefore, we defined a type 2 snore map consists of low- and mid-frequency snores and a type 3 snore map was composed of low- and high-frequency snores. Accordingly, we supposed that a higher grade of snore map typing may be resulted from a multi-site vibration in male OSAS patients. In our preclusive results, patients with snore map type 2 resembled patients with snore map type 1 in demographic and polysomnographic parameters (Table 2) although their snoring acoustic parameters differed to some extent (Table 3). The differences in clinical factors between the snore map type 3 and the snore map type 4 were insignificant despite some different acoustic parameters. Nevertheless, this classification system is aimed to illustrate the therapeutic targets of anti-snoring treatment.

Currently, there is no standard method for snoring acquisition. Therefore, some of those previous studies used ambient microphones placed at a specific location, some others used contact microphones or sensors usually placed at the trachea. In addition, the sampling frequencies and filtering bands were usually different. Accordingly, caution must be taken when comparing our results with the results of those other studies. Aside from those outlined above, this preliminary study has other limitations. Although this study enrolled patients with four different energy types of their snoring sounds, this snore map typing method should be further validated in the patients with primary snoring, and in the peer norms. Besides, the effect of nasal obstruction, a crucial factor of snoring [25], needs a further survey. The random error related to one-night studies should be further evaluated since this kind of snoring surveys are not reliable [26]. Moreover, the snore map measured the energy between 40 Hz and 2,000 Hz in mid-aged male OSAS patients. Higher frequency region of the snore sounds (>5,000 Hz) has been reported recently to be associated with OSAS snorers [27]. Accordingly, larger and more detail investigations will be useful to consider other important OSAS-related factors including age, gender, ethnicity, and high frequency snores. In addition, a prospective study is warranted to access the change of snore map types after anti-snoring treatment or OSAS therapy.

In conclusion, there are plenty of publications that have addressed the spectral analysis of snores, in subjects with and without OSAS. This study further supported the importance of analysis of the full-night snoring sounds. The highest intensity of the special-band snores (40 Hz–2,000 Hz or 851 Hz–2,000 Hz) and the mean frequency of total-frequency and high-frequency snoring sounds are reliable snoring sound parameters to predict the AHI among male OSAS patients; however, these predicators could not be measured by current subjective questionnaires appropriately. Snore map types can be classified easily and reliably, and are fairly related to the severity of OSAS. The clinical values of snore map typing such as differentiation of primary snoring and OSAS in larger populations and changes after OSAS treatment needs a further investigation.

## References

[1] Chuang LP, Hsu SC, Lin SW, Ko WS, Chen NH, et al (2008) Prevalence of snoring and witnessed apnea in Taiwanese adults. Chang Gung Med J 31: 175–181. 18567418

[2] Wilson K, Stoohs RA, Mulrooney TF, Johnson LJ, Guilleminault C, et al (1999) The snoring spectrum. Acoustic assessment of snoring sound intensity in 1,139 individuals undergoing polysomnography. Chest 115: 762–770. 10084490 10.1378/chest.115.3.762

[3] Fiz JA, Jané R, Solà-Soler J, Abad J, García MA, et al (2010) Continuous analysis and monitoring of snores and their relationship to the apnea-hypopnea index. Laryngoscope 120: 854–862. 20222022 10.1002/lary.20815

[4] Lim PVH, Curry AR (1999) A new method for evaluating and reporting the severity of snoring. J Laryngol Otol 113: 336–340. 10474668 10.1017/s0022215100143919

[5] Gliklich RE, Wang PC (2002) Validation of the snore outcomes survey for patients with sleep-disordered breathing. Arch Otolaryngol Head Neck Surg 128: 819–824. 12117343 10.1001/archotol.128.7.819

[6] Douglas SA, Webster S, El Badawey MR, Drinnan M, Matthews JN, et al (2006) The development of a snoring symptoms inventory. Otolaryngol Head Neck Surg 134: 56–62. 16399181 10.1016/j.otohns.2005.09.006

[7] Herzog M, Schmidt A, Bremert T, Herzog B, Hosemann W, et al (2008) Analysed snoring sounds correlate to obstructive sleep disordered breathing. Eur Arch Otorhinolaryngol 265: 105–113. 17680262 10.1007/s00405-007-0408-8

[8] Hoffstein V, Mateika S, Nash S (1996) Comparing perceptions and measurements of snoring. Sleep 19: 783–789. 9085486

[9] Chen NH, Li HY, Gliklich RE, Chu CC, Liang SC, et al (2002) Validation assessment of the Chinese version of the Snore Outcomes Survey. Qual Life Res 11: 601–607. 12206581 10.1023/a:1016337008763

[10] Cathcart RA, Hamilton DW, Drinnan MJ, Gibson GJ, Wilson JA (2010) Night-to-night variation in snoring sound severity: one night studies are not reliable. Clin Otolaryngol 35: 198–203. 20636738 10.1111/j.1749-4486.2010.02127.x

[11] Xu H, Huang W, Yu L, Chen L (2010) Sound spectral analysis of snoring sound and site of obstruction in obstructive sleep apnea syndrome. Acta Otolaryngol 130: 1175–1179. 20377505 10.3109/00016481003694774

[12] Yu JF, Chen YS, Li HY (2012) The characteristics of snoring at pharyngeal anatomy in natural sleep: snoring duration. J Mech 28: 91–95.

[13] Friedman M, Ibrahim H, Joseph NJ (2004) Staging of obstructive sleep apnea/hypopnea syndrome: a guide to appropriate treatment. Laryngoscope 114: 454–459. 15091218 10.1097/00005537-200403000-00013

[14] Li HY, Wang PC, Lee LA, Chen NH, Fang TJ (2006) Prediction of uvulopalatopharyngoplasty outcome: anatomy-based staging system versus severity-based staging system. Sleep 29: 1537–1541. 17252884 10.1093/sleep/29.12.1537

[15] Iber C, Ancoli-Israel S, Chesson AL, Quan SF (2007) The AASM manual for the scoring of sleep and associated events: Rules, terminology and technical specifications. 1^st^ ed. Westchester: American Academy of Sleep Medicine.

[16] White JE, Smithson AJ, Close PR, Drinnan MJ, Prichard AJ, et al (1994) The use of sound recording and oxygen saturation in screening snorers for obstructive sleep apnoea. Clin Otolaryngol Allied Sci 19: 218–221. 7923843 10.1111/j.1365-2273.1994.tb01218.x

[17] Jury EI, Blanchard J (1961) A stability test for linear discrete systems in table form. IRE Proc 49: 1947–1948.

[18] Perez-Padilla JR, Slawinski E, Difrancesco LM, Feige RR, Remmers JE, et al (1993) Characteristics of the snoring noise in patients with and without occlusive sleep apnea. Am Rev Respir Dis 147: 635–644. 8442599 10.1164/ajrccm/147.3.635

[19] Beck R, Odeh M, Oliven A, Gavriely N (1995) The acoustic properties of snores. Eur Respir J 8: 2120–2128. 8666109 10.1183/09031936.95.08122120

[20] Solà-Soler J, Jané R, Fiz JA, Morera J (2003) Spectral envelope analysis in snoring signals from simple snorers and patients with obstructive sleep apnea. Conf Proc IEEE Eng Med Biol Soc 3: 2527–2530.

[21] Ben-Israel N, Tarasiuk A, Zigel Y (2012) Obstructive apnea hypopnea index estimation by analysis of nocturnal snoring signals in adults. Sleep 35: 1299–1305. 22942509 10.5665/sleep.2092 PMC3413808

[22] Mesquita J, Solà-Soler J, Fiz JA, Morera J, Jané R (2012) All night analysis of time interval between snores in subjects with sleep apnea hypopnea syndrome. Med Biol Eng Comput. 2012 50: 373–381. 10.1007/s11517-012-0885-9 PMC3314810 22407477

[23] Miyazaki S, Itasaka Y, Ishikawa K, Togawa K (1998) Acoustic analysis of snoring and the site of airway obstruction in sleep related respiratory disorders. Acta Otolaryngol Suppl 537: 47–51. 9870649

[24] Agrawal S, Stone P, McGuinness K, Morris J, Camilleri AE (2002) Sound frequency analysis and the site of snoring in natural and induced sleep. Clin Otolaryngol Allied Sci 27: 162–166. 12071989 10.1046/j.1365-2273.2002.00554.x

[25] Li HY, Lee LA, Wang PC, Chen NH, Lin Y, et al (2008) Nasal surgery for snoring in patients with obstructive sleep apnea. Laryngoscope 118: 354–359. 18000468 10.1097/MLG.0b013e318158f73f

[26] Cathcart RA, Hamilton DW, Drinnan MJ, Gibson GJ, Wilson JA (2010) Night-to-night variation in snoring sound severity: one night studies are not reliable. Clin Otolaryngol 35: 198–203. 20636738 10.1111/j.1749-4486.2010.02127.x

[27] Emoto T, Abeyratne UR, Akutagawa M, Konaka S, Kinouchi Y (2011) High frequency region of the snore spectra carry important information on the disease of sleep apnoea. J Med Eng Technol 35: 425–431. 22066466 10.3109/03091902.2011.626838

